# The insulin resistant brain: impact on whole-body metabolism and body fat distribution

**DOI:** 10.1007/s00125-024-06104-9

**Published:** 2024-02-16

**Authors:** Martin Heni

**Affiliations:** 1 Division of Endocrinology and Diabetology, Department of Internal Medicine 1, University Hospital Ulm, Ulm, Germany; 2 Department for Diagnostic Laboratory Medicine, Institute for Clinical Chemistry and Pathobiochemistry, University Hospital of Tübingen, Tübingen, Germany

**Keywords:** Brain, Diabetes, Insulin, Insulin resistance, Obesity, Prediabetes, Review

## Abstract

Insulin exerts its actions not only on peripheral organs but is also transported into the brain where it performs distinct functions in various brain regions. This review highlights recent advancements in our understanding of insulin’s actions within the brain, with a specific emphasis on investigations in humans. It summarises current knowledge on the transport of insulin into the brain. Subsequently, it showcases robust evidence demonstrating the existence and physiological consequences of brain insulin action, while also introducing the presence of brain insulin resistance in humans. This pathophysiological condition goes along with an impaired acute modulation of peripheral metabolism in response to brain insulin action, particularly in the postprandial state. Furthermore, brain insulin resistance has been associated with long-term adiposity and an unfavourable adipose tissue distribution, thus implicating it in the pathogenesis of subgroups of obesity and (pre)diabetes that are characterised by distinct patterns of body fat distribution. Encouragingly, emerging evidence suggests that brain insulin resistance could represent a treatable entity, thereby opening up novel therapeutic avenues to improve systemic metabolism and enhance brain functions, including cognition. The review closes with an outlook towards prospective research directions aimed at further elucidating the clinical implications of brain insulin resistance. It emphasises the critical need to establish feasible diagnostic measures and effective therapeutic interventions.

**Graphical Abstract:**

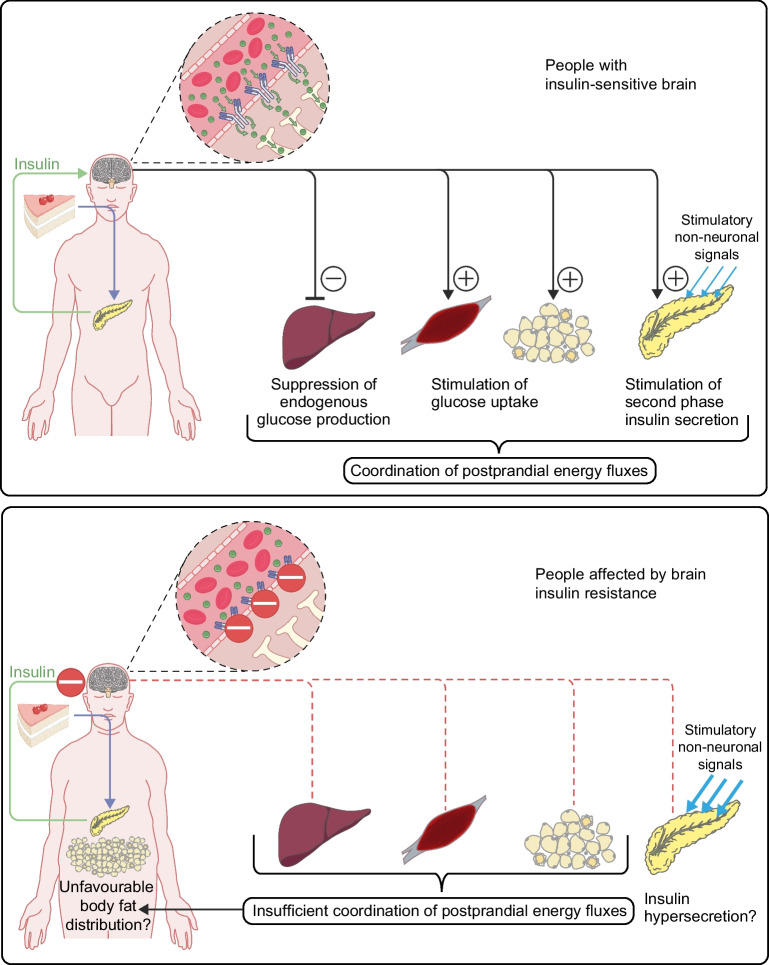

**Supplementary Information:**

The online version contains a slideset of the figures for download available at 10.1007/s00125-024-06104-9.

## Historical background: the discovery of brain insulin action and its contribution to systemic metabolism

The discovery of the brain’s role in whole-body metabolism goes back to the work of the French physiologist Claude Bernard in the mid-19th century. He discovered that puncturing the floor of the fourth ventricle in rabbits triggered glucosuria and resulted in the animals’ rapid demise, leading him to conclude that manipulating the brain can cause diabetes.

This foundational work was complemented by the discovery of insulin in 1921 and the subsequent discovery that it crosses the blood–brain barrier (BBB) in dogs [1]. The implications of this finding remained elusive, as insulin does not stimulate glucose uptake in neurons. Seminal works by Roth and colleagues in the mid-20th century demonstrated the presence of insulin receptors throughout the brain [2]. This finding prompted numerous experiments that revealed a complex interplay between the brain and systemic metabolism. The groundbreaking work of Woods and colleagues emphasised the pivotal role of brain insulin action, revealing that direct administration of insulin into the brain attenuated food intake and reduced body weight in a number of species, including baboons [3–5]. In line, brain-specific insulin-receptor knockout in rodents increased food intake and body weight, and induced systemic insulin resistance as well as hypertriglyceridaemia [6]. Another breakthrough was the discovery that brain insulin could modulate glucose metabolism via its influence on hepatic glucose production [7], presumably via the parasympathetic nervous system [8].

Subsequently, numerous studies characterised how brain insulin modulates whole-body glucose and lipid metabolism, and how it regulates appetite, energy expenditure and body weight. Through this scientific journey, brain insulin was discovered to impact multiple further brain functions such as cognition, memory and synaptic plasticity. This review mainly focuses on the role of brain insulin in human metabolism.

## Transport of insulin into the human brain

The BBB is a selective, semi-permeable barrier around the microvasculature in the brain. It plays a crucial role in brain health by strictly controlling molecular passage between the bloodstream and the brain [9, 10]. The primary (but likely not the only) mechanism enabling insulin to enter the brain is receptor-mediated transcytosis [9–11] (i.e. binding to insulin receptors on the BBB and then moving across endothelial cells into the brain’s extracellular space [9, 10, 12]). In certain specialised brain regions, such as the arcuate nucleus of the hypothalamus, the BBB appears to be less dense [10]. Here, tanycytes, a cell type also expressing the insulin receptor [13], are required for insulin uptake [14].

Similar to animals [1], insulin is present in the cerebrospinal fluid (CSF) of humans [15]. This compartment is accessible for investigations of transport processes into the brain. Presence of insulin in the human CSF indicates that the hormone is transported from the bloodstream into the central nervous system. However, findings are still controversial as to whether tiny amounts of insulin may also be produced locally [16].

Insulin transport is not equally effective in all individuals. There appear to be a number of situations that either facilitate or hinder this transport, ultimately influencing insulin availability within the brain: insulin penetration into the CSF is lower in individuals with obesity [17, 18]. Furthermore, alterations in blood glucose levels acutely modulate the transport of peptide hormones, including insulin, into the CSF [18]. Thus, alterations in the transportation process across the BBB that requires the insulin receptor could account for the observed variations in insulin transport into the human CSF. In line, diminished transportation efficiency has been identified in individuals who present with systemic insulin resistance [19].

Ageing is also linked to a reduction in insulin transport into the CSF [20]. This decline may contribute to compromised brain insulin action, predisposing individuals to age-related cognitive dysfunction and neurodegenerative diseases [21]. This is supported by reduced CSF insulin concentrations in individuals with Alzheimer’s disease in some [22–24] but not all studies [24]. Yet, the precise underlying mechanisms governing the regulation of insulin transport into the human brain remain under investigation [11].

## Evidence for brain insulin action and the existence of brain insulin resistance in humans

### Evidence for brain insulin action

Just as in animals, insulin receptors are expressed in the human brain in neurons and other cell types (e.g. astrocytes) [12, 25, 26], teleologically arguing for a role of insulin in the brain.

Various techniques are used to stimulate brain insulin action in clinical research. The most physiological way is to measure the response to endogenous insulin that is released in response to food intake. However, numerous additional postprandial factors [27] hinder the dissection of insulin’s specific effects from other effects. A more selective approach is the i.v. infusion of insulin during hyperinsulinaemic–euglycaemic glucose clamps. However, this technique also cannot differentiate between peripheral and brain effects. One approach frequently used in clinical research to overcome this challenge is the administration of insulin by nasal spray [28]. This route delivers a substantial amount of insulin to the brain [29], while only small amounts enter the bloodstream [30, 31]. The quantity of insulin absorbed into the bloodstream is not sufficient to induce hypoglycaemia [30–32] and likely does not significantly contribute to the induced brain effects [31, 32]. Nevertheless, this insulin spillover must be taken into account when studying the potential impact of brain insulin on peripheral metabolism.

Modern neuroimaging techniques, such as functional MRI (fMRI), positron emission tomography (PET) and magnetoencephalography (MEG), have facilitated investigations into the effects of insulin on brain functions. MEG measures magnetic fields produced by the brain’s electrical activity. Early studies employing MEG demonstrated insulin’s impact on neuronal activity [33] and linked brain insulin effects to body weight [33, 34], metabolic factors [35] and genetic factors [36–38]. PET allows the assessment of metabolic processes, and most studies investigating insulin’s effects on the brain have employed the tracer fluorodeoxyglucose (FDG) to measure brain glucose uptake under insulin stimulation [39]. One study utilised the tracer raclopride to assess insulin’s effects on dopamine receptor availability [40].

Most studies on insulin action in the human brain have employed fMRI. In contrast to MEG, it provides higher spatial resolution for not only cortical but also subcortical regions. MRI enables detailed imaging of the brain’s anatomical structure and fMRI can also quantify functional aspects. With this technique, insulin-induced changes in regional brain activity and brain networks were detected [41]. Insulin-responsive networks and regions include areas critical for energy metabolism, eating behaviour, reward processes, mood and cognitive functions [25, 31, 41]. A recent systematic review of 58 RCTs using fMRI reported significant insulin effects in the inferior and middle frontal gyri, the dorsal striatum, the insula and the hypothalamus [31]. Further effects were reported in subcortical areas, including the hippocampus, in some but not all studies [31]. The insulin-responsive frontal gyri are part of the prefrontal cortex, which is involved in various high-level cognitive functions, including decision making and inhibitory control [31, 41, 42]. The dorsal striatum plays a crucial role in the brain’s reward system [40, 41, 43]. Its complex responses to insulin appear to contribute to the brain-derived modulation of peripheral insulin sensitivity [40, 44]. Notably, insulin modulates the tone of the principal neurotransmitter dopamine within this specific region of the human brain [40]. The insula is implicated in a wide range of functions [45] and plays a significant role in regulating the body’s homeostasis. Moreover, it is involved in the perception of bodily states, such as hunger and fullness [45], making it also essential for eating behaviour [31, 41]. The hypothalamus consists of various nuclei, some of which are critical for whole-body energy homeostasis, eating behaviour and body weight [46].

Hence, combining fMRI with nasal administration of insulin to assess insulin responses in regional cerebral blood flow in these areas could be a reliable and robust approach [31, 47] for quantifying brain insulin sensitivity in future trials.

### Brain insulin resistance in humans

Using the techniques described above, it has become clear that insulin affects human brain activity. However, there is a substantial number of people with reduced or even absent brain response to insulin, a state termed ‘brain insulin resistance’ [25, 31, 48]. This condition is most commonly associated with overweight and obesity [25]. Additionally, further factors are also linked to brain insulin resistance, including normal ageing [41, 49, 50], circulating levels of NEFA [35] and different common genetic polymorphisms [36–38, 51, 52], most of which were discovered due to their association with body weight. Though, the direct role of these factors in causing brain insulin resistance is still under research.

Furthermore, recent neuroimaging data suggest sex differences in brain responses to insulin [47, 53]. In young women, insulin sensitivity of the hypothalamus appears to be rapidly modulated across the menstrual cycle with relative insulin resistance in the luteal phase [47]. However, not all studies on insulin action in the human brain report sex differences and the potential underlying mechanisms remain largely unexplored.

Further evidence for the effects of insulin in the human brain comes from functional studies, where nasal administration of insulin improved memory, altered eating behaviour and affected mood, at least in certain populations. These functions have been reviewed in greater detail elsewhere [28, 31]. In line with findings from neuroimaging studies, there are data suggesting sex differences in the effects of acute intranasal insulin delivery on eating behaviour and memory functions [28].

## Effects of brain insulin action on peripheral metabolism

The first reports on genetic manipulation of brain insulin action in rodents suggested profound effects on peripheral metabolism [6]. Subsequent research in humans indicated that brain insulin action has a similar impact on the periphery, at least in individuals who are healthy and lean [25]. However, the precise mechanisms of signal transduction and regulation at the cellular level are still largely unexplored in humans.

A variety of clinical trials explored the metabolic effects of either nasal administration of insulin to the brain [44, 54–58] (for an overview see also electronic supplementary material [ESM] Tables 1, 2) or the pharmacological inhibition of brain insulin action [59, 60]. These studies indicated that brain insulin action has the potential to improve peripheral insulin sensitivity [44, 54, 55, 58, 59]. In most studies, this enhancement started approximately 45 min after nasal administration of insulin and was observed for at least 3 h [44, 55]. This outcome seems to involve several key mechanisms.

Brain insulin action suppresses endogenous glucose production [44, 56, 59], although the precise mechanisms and relative contribution is still under investigation (for review see, e.g. [61, 62]). In humans, this function seems only to occur under systemic hyperinsulinaemia but not at fasting insulin levels [63, 64]. Therefore, brain insulin might not directly inhibit hepatic glucose production, but rather enhance hepatic insulin sensitivity. This would facilitate the suppression of endogenous glucose production after meals when circulating insulin levels are high and insulin signalling in the brain occurs. In addition, brain insulin also acutely enhances liver energy metabolism and reduces liver fat content [64], presumably by promoting hepatic VLDL export (for review see, e.g. [65]). However, chronic intranasal insulin treatment did not change liver fat content but instead enhanced the liver’s secretion of branched-chain amino acids [57].

The impact of brain insulin action on human lipolysis is still not fully clear. While early studies suggested a suppressive effect [66], later trials that tightly controlled circulating insulin found no impact [64, 67, 68]. Considering the potent lipolysis-suppressing effect of even small increases in circulating insulin, it seems unlikely that brain-derived signals could substantially influence postprandial lipolysis under physiological circumstances with concurrently elevated insulin in both the circulation and the brain. Furthermore, brain insulin action appears to stimulate peripheral glucose uptake [44], thereby also contributing to improved peripheral insulin sensitivity (possible underlying mechanisms reviewed, e.g. in [61]).

Besides these effects on glucose handling, brain insulin acutely enhances glucose-stimulated insulin secretion from the pancreas [69]. Of note, this effect is exclusive for the second phase of insulin secretion and is closely linked to intact hypothalamic insulin sensitivity [69]. Mechanisms are likely similar to those underlying cephalic insulin responses [70, 71] and will therefore rely on the dense innervation of pancreatic islets [72]. While insulin resistance in the hypothalamus seems to hinder the acute stimulation of pancreatic insulin release, it was found to be paradoxically associated with insulin hypersecretion in response to oral glucose load in a cross-sectional study [73]. One possible explanation could be a long-term impairment of pancreatic inputs from the brain, as occurs in states of obesity and hypothalamic insulin resistance, which disrupts the balance of inputs to the beta cell. This imbalance could lead to an overabundance of stimulatory non-neuronal signals that promote insulin hypersecretion. Indeed, this has been observed in individuals with hypothalamic lesions [74] and may also occur in those with hypothalamic insulin resistance.

### Possible impact of brain insulin resistance

Importantly, all these observations on the regulation of whole-body metabolism by brain insulin were made solely in lean individuals. These effects are diminished or absent in people who are overweight or obese or who have brain insulin resistance or type 2 diabetes (as reported for brain insulin effects on peripheral insulin sensitivity [44, 55], endogenous glucose production [44, 75], peripheral glucose uptake [44], liver energy metabolism [64], liver fat content [64] and pancreatic insulin secretion [69]). It is still unclear whether obesity itself or the often associated whole-body insulin resistance is responsible, as there are no studies on brain insulin action in individuals who are obese but still insulin sensitive. Longitudinal studies are needed to determine the sequence of development between peripheral and brain insulin resistance.

### Potential (patho)physiological role of brain insulin in whole-body metabolism

**Fig. 1:**
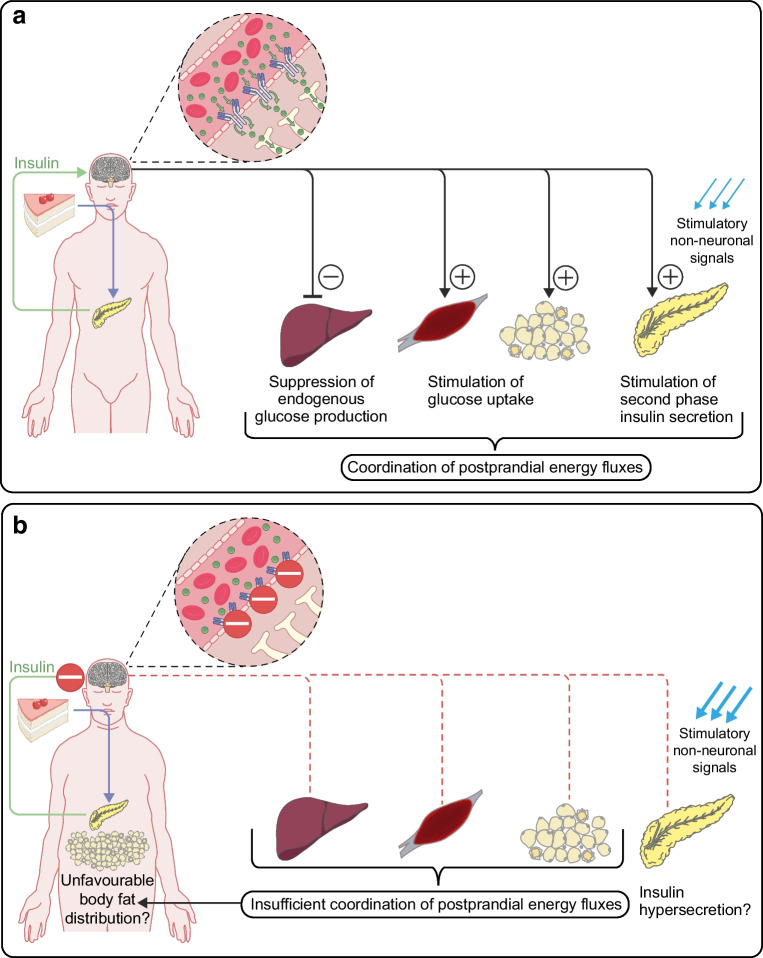
Putative model of brain insulin’s role in peripheral metabolism and the impact of brain insulin resistance. (**a**) Presumed situation in people with an insulin-sensitive brain. Upon food intake, insulin is released from the pancreas into the bloodstream. It reaches the brain, passes the BBB in a receptor-mediated process and activates specialised neurons (e.g. in the hypothalamus). This introduces signals to the pancreas that propagate second-phase insulin secretion. More insulin is released into the portal vein and acts as a strong suppressor of hepatic glucose production. Endogenous glucose production is further suppressed by direct signals from the brain to the liver. This mechanism likely contributes to the adequate suppression of hepatic glucose output after food intake and all-together coordinates energy fluxes throughout the organism. (**b**) Presumed situation in people affected by brain insulin resistance. In this scenario, insulin cannot properly pass the BBB and cannot properly activate specialised neurons in the brain. Signals towards the periphery are compromised. Hence, there is no acute stimulation of pancreatic insulin secretion through brain-derived signals. Of note, chronic lack of these regulatory signals could contribute to insulin hypersecretion due to an overabundance of stimulatory non-neuronal signals. Furthermore, in brain insulin resistance, signals towards the liver and other metabolic organs are lacking. Altogether, this could contribute to an impaired suppression of hepatic glucose output in the postprandial state and to an impaired brain-derived modulation of whole-body energy fluxes. Over time, this could facilitate an unfavourable body fat distribution with visceral obesity, a key phenotype of high-risk subgroups of diabetes and prediabetes. This figure is available as part of a downloadable slideset

**Fig. 2:**
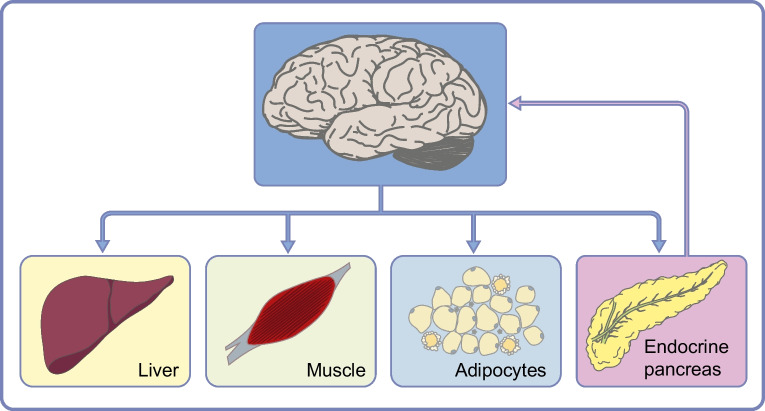
Overview of brain insulin action derived effects on peripheral metabolism. In response to food intake, insulin is released into the bloodstream. After passing the BBB, insulin reaches the brain where it acts in specialised areas, including the hypothalamus, frontal areas, insula and the dorsal striatum. This induces signals towards the periphery to suppress hepatic glucose production, to enhance peripheral glucose uptake into tissues (e.g. skeletal muscle and adipose tissue) and to propagate second-phase insulin secretion from the pancreas. These functions appear to be disturbed in brain insulin resistance. This figure is available as part of a downloadable slideset

Based on the above-described findings, we suggest a model for brain insulin’s role in peripheral glucose metabolism [25] (summarised in Fig. 1). Food intake triggers pancreatic insulin release, which crosses the BBB and reaches the brain. Here, it acts on specific neurons (e.g. in the hypothalamus). This triggers brain-derived signals to metabolic organs in the periphery, enhancing liver insulin sensitivity and boosting pancreatic insulin secretion into the portal vein, further stimulating hepatic insulin action. These processes together contribute to an effective suppression of hepatic glucose production. At the same time, brain-derived signals promote glucose uptake into peripheral tissues. Altogether, this ensures proper synchronisation of energy handling in various metabolic organs in the periphery in the postprandial state (Fig. 2).

### Signals from the brain to metabolic organs in the periphery

Brain-to-periphery signals are likely transmitted through the autonomic nervous system [25, 70, 72, 76]. In line, brain insulin action appears to promote a transition from sympathetic to parasympathetic dominance [32, 55, 77] that suppresses endogenous glucose production and stimulates pancreatic insulin secretion [78]. Further signalling pathways may exist, potentially involving circulating factors, although traditional endocrine systems (e.g. hypothalamic–pituitary–adrenal axis) appear unaffected by the acute effects of brain insulin [32, 40, 54, 79].

In brain insulin resistance, the brain’s acute modulation of peripheral metabolism seems to be disrupted [25], possibly leading to impaired or absent brain-derived coordination of postprandial energy distribution in the body.

The relative contribution of brain-derived modulation to human postprandial glucose metabolism, compared with direct effects on peripheral organs, remains an intriguing question. The observations that nasal administration of insulin reduces the need for endogenous insulin post-meal [58] and that long-term nasal administration of lower-dose insulin decreases glucose fluctuations [80], suggest a significant role for brain insulin action in physiological glucose regulation. However, the reduced glucose excursions in the later study did not translate into reduced HbA_1c_ levels [81]. Thus, it is still uncertain whether, and to what extent, disruptions in brain insulin circuits contribute to abnormal glucose metabolism (e.g. in type 2 diabetes).

## Long-term consequences of brain insulin resistance in humans

The majority of data on the potential impact of brain insulin resistance comes from cross-sectional analyses that suggest connections to visceral obesity and metabolic diseases [25], as well as neurological and psychiatric disorders. These include neurodegeneration, Alzheimer’s disease, Parkinson’s disease and depression (reviewed in greater detail in [48, 82, 83]). However, the predictive power of these associations is restricted due to a limited number of longitudinal studies.

In line with findings from cross-sectional studies [84–86], brain insulin sensitivity is longitudinally associated with future body weight and body fat distribution [86–88]. Intriguingly, brain insulin sensitivity before a 24 month lifestyle intervention programme aiming to prevent type 2 diabetes predicted the programme’s effectiveness [86, 87]. Participants with brain insulin resistance struggled to lose weight or reduce their visceral fat, whereas those with good brain insulin responsiveness achieved substantial benefits. Long-term follow-up data collected 9 years afterwards indicated a lasting impact on body weight and body fat distribution, with unfavourable courses in those with brain insulin resistance [86]. Similarly, a recent trial found that brain insulin responsiveness predicted weight loss success in response to a 3 month caloric restriction in overweight, metabolically healthy adults [88].

Recent research in people at increased risk for type 2 diabetes based on detailed phenotyping has identified six unique clusters (i.e. six distinct groups of people with similar phenotypic characteristics) [89, 90]. Three clusters have a heightened risk of developing diabetes and varying risks of nephropathy, CVD and all-cause mortality, independent of blood glucose. Of note, the risk of complications is only partially connected to diabetes risk [89]. While the mechanisms driving these high-risk phenotypes remain largely unexplored, it is noteworthy that the phenotype of individuals at high risk for complications closely resembles that seen in individuals with brain insulin resistance. Therefore, brain insulin resistance may potentially be a critical factor in the pathogenesis of a phenotype with a high risk for complications of prediabetes and diabetes, a hypothesis that needs to be tested in upcoming studies.

To my knowledge, no dedicated longitudinal studies have been conducted that explore the impact of brain insulin resistance on cognitive function and mood. Nevertheless, numerous studies have investigated the impact of whole-body insulin resistance, which often overlaps with brain insulin resistance. These suggest predictive links between insulin resistance and accelerated cognitive decline [91], Alzheimer’s disease [21, 48], Parkinson’s disease [48, 82] and depression [92–94]. It is still being investigated whether insulin resistance is the pathomechanism or whether a common element like a proinflammatory state induces both insulin resistance and brain diseases.

## Treatment of brain insulin resistance

Given the far-reaching implications on cognitive, neurological and metabolic health, brain insulin resistance is a compelling target for therapeutic intervention. However, human studies are scarce.

Two recent clinical trials that quantified brain responses to nasal insulin by fMRI demonstrated that brain insulin resistance appears to be a treatable condition. The first trial included young individuals who were overweight or obese. Despite no significant weight loss, an 8 week exercise intervention improved insulin responsiveness in the dorsal striatum (putamen) to a level similar to that seen in lean individuals [95]. The second trial evaluated pharmacological treatment with the sodium–glucose cotransporter 2 (SGLT2) inhibitor empagliflozin in individuals with prediabetes who were overweight or obese [96]. Irrespective of weight loss, SGLT2 inhibition over 8 weeks restored hypothalamic insulin sensitivity. Of note, mediation analyses indicated that this improvement in hypothalamic insulin responsiveness appears to drive the reduction of liver fat content and enhancement of fasting blood glucose levels that were also achieved with this SGLT2 inhibitor treatment [96].

In line with these findings, brain effects have been reported for dapagliflozin, another SGLT2 inhibitor [97]. While this study was focused on food-cue reactivity and did not test brain insulin responsiveness, it appears likely that empagliflozin and dapagliflozin have comparable effects [98]. While SGLT2 is expressed in the brain [99], it is unclear whether these pharmacological inhibitors act there directly or indirectly via peripheral action with subsequent projections towards the brain [98, 99].

Thiazolidinediones, a class of insulin-sensitising drugs, have not been specifically evaluated for their impact on brain insulin responsiveness in humans. Some studies have explored their effects on cognitive functions, primarily focusing on Alzheimer’s disease, yielding mixed results [21, 100]. The latest large RCT with pioglitazone was terminated early due to ineffectiveness [101]. Known side effects and the lack of cognitive benefits in dementia suggest limited potential of this substance class for treating brain insulin resistance.

Current large-scale studies are assessing the effects of glucose-lowering medications such as SGLT2 inhibitors and GLP-1 receptor agonists in neurological diseases, and may shed light on new pharmacological treatments for brain insulin resistance.

Even after significant weight loss through bariatric surgery, insulin’s effects on brain glucose uptake, as evaluated using FDG PET-CT, were not brought back to what is observed in lean individuals [102]. However, a recent study using fMRI with nasal insulin demonstrated improved brain insulin responsiveness after a 3 month low-energy diet [88]. Both imaging approaches likely capture different features of brain insulin action, highlighting the need for further research to clarify the effects of bariatric surgery and weight loss on brain insulin responsiveness.

Although evidence is growing that brain insulin resistance is in principle treatable, larger, randomised trials are necessary to confirm this and to clarify its clinical significance.

## Future research directions

Even with a mounting body of evidence supporting the clinical significance of insulin action within the human brain, many open questions still remain. One of the key challenges is understanding the complex communication between the brain and peripheral organs. Unravelling the extent to which insulin-induced effects in the brain contribute to the regulation of whole-body metabolism following food intake, compared with the direct effects of insulin on target organs such as the liver or adipocytes, will be an intriguing endeavour. Clearly, more research in this direction and new non-invasive tools are necessary to decipher the mechanisms that underly each crucial step of the pancreas–brain–periphery network.

Another challenge is to uncover the regulatory processes governing insulin’s transport into the brain, as well as the precise mechanisms through which signals originating from the brain are conveyed towards target organs. Understanding whether the regulation of these mechanisms varies for each target is essential for a comprehensive understanding of the system as a whole as well as for developing organ-specific interventions.

Insulin signalling in the brain occurs physiologically in the postprandial state, a situation during which numerous signalling factors undergo dynamic fluctuations (e.g. incretin hormones and glucagon [27]). Furthermore, additional factors inform the brain about energy availability, including leptin. Determining how these factors interact with insulin in neurons and other brain cells is a largely unchartered territory and needs exploration. This will be of special importance as a number of upcoming pharmacotherapies specifically address such postprandial signalling pathways.

Further exploration is needed on how impaired brain insulin action contributes to high-risk phenotypes in prediabetes and diabetes, neurological disorders and psychiatric conditions. This could clarify the pathophysiological contribution, thereby aiding more effective prevention and intervention strategies.

A significant obstacle in assessing brain insulin resistance in larger studies and testing the potential role in clinical management is the lack of precise biomarkers. Current diagnostic procedures, such as fMRI combined with nasal administration of insulin, are costly and time-consuming. Developing easy-to-use, non-invasive tools such as biomarkers, digital tools or combinations thereof should become a priority. Such advancements could simplify diagnoses, enable accurate risk stratification and facilitate monitoring of disease progression.

Lastly, refining and optimising therapeutic methods for brain insulin resistance could open preventative or therapeutic possibilities not only for obesity and metabolic disorders but also for related neurological and psychiatric conditions.

In conclusion, exploring the role of brain insulin signalling is a thrilling and rapidly evolving research field, with implications beyond glucose metabolism. Progress in this central area requires a multidisciplinary effort to translate research findings into clinical practice and improve people’s lives.

### Supplementary Information

Below is the link to the electronic supplementary material.

Supplementary file1 (PDF 231 KB)

Slideset of figures (PPTX 390 KB)

## Abbreviations

BBB: Blood–brain barrier
CSF: Cerebrospinal fluid
FDG: Fluorodeoxyglucose
fMRI: Functional MRI
MEG: Magnetoencephalography
PET: Positron emission tomography
SGLT2: Sodium–glucose cotransporter 2
