# Supplementary material for: The insulin resistant brain: impact on whole-body metabolism and body fat distribution
Source: Diabetologia. 2024 Feb 16;67(7):1181–91. doi: 10.1007/s00125-024-06104-9 (PMC11153284; doi:10.1007/s00125-024-06104-9)
Supplement: Supplementary file 1 — Supplementary file1 (PDF 231 KB) [file 125_2024_6104_MOESM1_ESM.pdf]

**ESM table 1: Summary of acute effects of intranasal insulin on systemic metabolism**

| | |
|---|---|
| **Effect on peripheral glucose metabolism / insulin sensitivity** | |
| In lean healthy persons | • Improvement: [1–5]<br>• No detectable effect: [6] |
| In persons with overweight or obesity | No detectable effect: [1, 4] |
| In persons with type 2 diabetes | No data available |
| | |
| **Effects on endogenous glucose production** | |
| In lean healthy persons | • Suppression under systemic hyperinsulinemia: [1]<br>• Suppression under fasting insulin concentrations: [7]<br>• No detectable effect under fasting insulin concentrations: [8, 9] |
| In persons with overweight or obesity | • No detectable effect under systemic hyperinsulinemia: [1]<br>• No detectable effect under fasting insulin concentrations: [10] |
| In persons with type 2 diabetes | • No detectable effect under fasting insulin concentrations: [8] |
| | |
| **Effects on pancreatic insulin secretion** | |
| In lean healthy persons | Stimulation: [11, 12] |
| In persons with overweight or obesity | No data available |
| In persons with type 2 diabetes | No data available |
| | |
| | |
| **Effect on circulating free fatty acids** | |
| In lean healthy persons | • Suppression: [13]<br>• No detectable effect: [1, 5, 8, 14] |
| In persons with overweight or obesity | No detectable effect: [1] |
| In persons with type 2 diabetes | No detectable effect: [8] |
| | |
| **Effect on food intake** | |
| In lean healthy persons | • Suppression: [15–19]<br>• No detectable effect: [18, 20, 21]15/01/2024 18:46:00 |
| In persons with overweight or obesity | • Suppression: [21]<br>• No detectable effect: [22] |
| In persons with type 2 diabetes | No data available |
| | |
| **Effect on endocrine systems** | |
| In lean healthy persons | • Effects: [23–25]<br>• No detectable effect: [26–29] |
| In persons with overweight or obesity | No data available |
| In persons with type 2 diabetes | No detectable effect: [28] |

**ESM table 2: Studies on acute effects of brain insulin delivery as nasal spray in humans**

| First author | Study population | Dose and type of nasal insulin | Main findings |
|---|---|---|---|
| Kern et al. [30] | 18 healthy males | 120 U human insulin vs placebo | Changes in auditory evoked potentials, no effect on norepinephrine levels |
| Born et al. [31] | 8 healthy males | 40 U human insulin vs placebo | Increase in cerebrospinal fluid insulin, no effect on serum insulin |
| Reger et al. [32] | 26 subjects with impaired memory and 35 healthy subjects | 20 U or 40 U human insulin or placebo (cross-over) | APOE-dependent improved verbal memory in memory-impaired group |
| Benedict et al. [15] | 32 normal-weight persons (18 females) | 160 U human insulin versus placebo once (cross-over) | Decreased food intake in men, but not in women. Improved memory in women, but not in men |
| Bohringer et al. [33] | 26 healthy young males | 40 U human insulin versus placebo once (randomized parallel) | Diminished cortisol response to the Trier social stress test |
| Reger et al. [34] | 59 controls and 33 patients with impaired memory | 10, 20, 40, 60 U human insulin versus placebo once (cross-over) | Improved verbal memory, peak effect with 20 U, no effect on plasma insulin or blood glucose |
| Guthoff et al. [35] | 9 healthy persons (5 males) | 160 U human insulin versus placebo once (cross-over) | Altered processing of food pictures (assessed by fMRI) |
| Krug et al. [36] | 14 healthy postmenopausal women | 160 U human insulin versus placebo once (cross-over) | No effect on food intake, enhanced memory |
| Stingl et al. [37] | 10 lean/10 overweight healthy volunteers | 160 U human insulin once versus placebo (cross-over) | Changes in resting state dynamics (assessed by MEG) |
| Benedict et al. [38] | 19 healthy men | 160 U human insulin versus placebo once (cross-over) | Enhanced postprandial thermogenesis, lower postprandial circulating insulin and C-peptide levels |
| Fan et al. [39] | 30 patients with schizophrenia | 40 U human insulin versus placebo once (randomized parallel) | No effect on verbal memory or sustained attention |

| | | | |
|---|---|---|---|
| Guthoff et al. [40] | 10 lean persons and 10 persons with obesity | 160 U human insulin versus placebo once (cross-over) | Altered cerebral processing of food pictures in lean, but not obese (assessed by MEG) |
| Stockhorst et al. [41] | 32 healthy young persons | 20 U human insulin once (randomized parallel) | Conditioned increase in peripheral insulin |
| Stein et al. [42] | 32 patients with mild to moderate Alzheimer´s disease (16 females treated with nasal insulin) | 60 U human insulin (n=16) versus placebo (n=16) 4 times daily for 2 days (total 480 U insulin) (parallel-design) | No effect on memory |
| Grichisch et al. [43] | 8 healthy persons (3 males) | 160 U human insulin versus oral caffeine (200mg) once (cross-over) | No effect on global cerebral blood flow, i.e. no direct vasodilatory effect of nasal insulin (assessed by fMRI) |
| Hallschmid et al. [44] | 30 healthy women | 160 U human insulin versus placebo once (randomized parallel) | Decreased postprandial appetite, decreased postprandial intake of chocolate cookies |
| Heni et al. [3] | 103 lean volunteers (68 females) | 160 U human insulin versus placebo once (cross-over) | Change in HOMA-IR and change in brain activity (assessed by fMRI), no effect on cortisol levels |
| Ketterer et al. [27] | 43 lean volunteers | 160 U human insulin versus placebo once (cross-over) | Reduced brain insulin responsiveness in minor allele carriers of *CNR* SNP rs3123554 (assessed by MEG) |
| Jauch-Chara et al. [45] | 15 healthy men | 40 U human insulin versus placebo once (cross-over) | Increased brain ATP and phosphocreatine levels (assessed by $^{31}$P MR spectroscopy) |
| Brünner et al. [3, 46, 47] | 14 healthy persons (7 females) | 40 U human insulin once versus placebo (cross-over) | Decrease in olfactory threshold |
| Kullmann et al. [48] | 17 female volunteers | 160 U human insulin once versus placebo (cross-over) | Modification of reward processes and prefrontal brain activity (assessed by fMRI) |

| | | | |
|---|---|---|---|
| Heni et al. [49] | 10 healthy lean men and 5 healthy men with obesity | 160 U human insulin once versus placebo during systemic hyperinsulinemia (cross-over) | Improved peripheral insulin sensitivity, modulation of hypothalamic activity (assessed by fMRI), change in heart rate variability. Effects only in lean, but not overweight men. |
| Iwen et al. [50] | 14 healthy men | 160 U human insulin once versus placebo (cross-over) | Decrease in circulating free fatty acids and lipolysis |
| Novak et al., 2014; Zhang et al. [51, 52] | 15 patients with type 2 diabetes, 14 controls | 40 U human insulin once versus saline (randomized parallel) | Improvement in cognitive function, change in cerebral blood flow (assessed by MRI) |
| Ferreira de Sá et al., Schilling et al. [53, 54] | 54 healthy volunteers | 40 U human insulin (n=13), 30 mg Cortisol (n=12), 30 mg Cortisol + 40 U human insulin (n=15), placebo (n=14) (parallel-design) | No effect of insulin on processing of food cues (assessed by fMRI) |
| Brünner et al. [51, 52, 55] | 18 male persons | 40 U human insulin once versus placebo (cross-over) | Improved delayed but not immediate odor-cue recall of spatial memory |
| Gancheva et al. [56] | 10 patients with type 2 diabetes (1 female), 10 healthy volunteers (3 females) | 160 U human insulin once versus placebo (cross-over) | Improvement in hepatic energy metabolism and decline in liver fat content in lean persons (assessed by MR spectroscopy) |
| Kullmann et al. [57–59] | 25 lean (10 female) and 23 (11 female) overweight healthy volunteers | 160 U human insulin once versus placebo (cross-over) | Change in regional brain activity (assessed by fMRI) |
| Ott et al. [6] | 20 healthy men | 10 and 20 U insulin aspart every 15 min over 5 hours (total dose 210 and 420 U) versus placebo (cross-over). Separate control group (N=10) with iv insulin aspart (0.12 U/kg/24 h) | No effects on glucose metabolism above what was observed in the control group with iv insulin aspart |

| | | | |
|---|---|---|---|
| Dash et al. [7] | 8 healthy men | 40 U insulin lispro once versus placebo (cross-over) during pancreatic clamp | Suppression of endogenous glucose production despite similar venous insulin concentrations to placebo condition |
| Schöpf et al. [60] | 10 patients with smell loss | 40 U human insulin (n=10), NaCl at later time point (n=7) once | Improved olfactory sensitivity and intensity |
| Feld et al. [61] | 16 healthy men and 16 healthy women | 160 U human insulin versus placebo (cross-over) | Increased growth hormone concentrations in the night-half following nasal insulin, impaired memory encoding on subsequent day |
| Brünner et al. [62] | 16 healthy men | 40 U human insulin versus placebo once (cross-over) | No effect of nasal insulin on declarative memory or hippocampal activity |
| Hamidovic et al. [63]; [64] | 19 healthy smokers (cross-over) and 37 healthy smokers (parallel) abstained from smoking for 36h | 60 U human insulin versus placebo once | Reduction in nicotine craving, increase in circulating cortisol during psychosocial stress.<br><br>No improvement of verbal learning memory in smokers. |
| Heni et al. [65] | 11 lean and 10 overweight healthy men | 160 U human insulin versus placebo once during systemic hyperinsulinemia (cross-over) | Improvement in peripheral insulin sensitivity by suppression of endogenous glucose production and stimulation of glucose uptake into tissue. Change in regional brain activity in hypothalamus and striatum (assessed by fMRI). Detailed characterization and mimicking of spillover of nasal insulin into circulation |

| | | | |
|---|---|---|---|
| Rodriguez-Raecke et al. [66] | 24 healthy males | 40 U human insulin versus placebo once (cross-over) | Improved gustatory sensitivity |
| Santiago and Hallschmid. [67] | 51 healthy (32 young and 19 elderly) men and women | 160 U human insulin versus placebo once (cross-over) | Reduced food intake, no effect on sleep patterns |
| Akintola et al. [68] | 19 adults (11 older and 8 young) | 40 U human insulin versus placebo (cross-over) | Improved brain perfusion in occipital lobe and thalamus in older persons (assessed by fMRI) |
| Thienel et al. [69] | 14 elderly and 30 young healthy persons | 160 U human insulin versus placebo once (cross-over) | Reduced cortisol levels in the night half following nasal insulin in elderly, but not in young participants |
| Opstal et al. [70] | 8 healthy, normal weight adult men | 40 U human insulin versus placebo (cross-over) | Enhanced effect of oral glucose ingestion on the hypothalamus (assessed by fMRI) |
| Kullmann et al. [71] | 9 healthy men | 40 U, 80 U, 160 U human insulin versus placebo (four-way cross-over) | Dose-depended effect on regional brain activity (assessed by fMRI) and on the autonomic nervous system. Detailed characterization of insulin spillover into systemic circulation. No effect on pituitary hormone axes. |
| Krug et al. [72] | 32 healthy men | 160 U human insulin versus placebo (cross-over) | Independent effects of nasal insulin and estradiol on macronutrient intake |
| Yokoyama et al. [71, 73] | 15 normal weight and 8 overweight men and women | 40 U human insulin versus saline (cross-over) | Diminished fat oxidation during exercise in overweight persons after insulin nasal spray |
| Dhindsa et al. [74] | 14 men (8 with T2DM, 6 healthy | 40 U human insulin versus Placebo (cross-over) | No acute changes in LH concentrations in men |

| | | | |
|---|---|---|---|
| Xiao et al. [10] | 7 men with overweight or obesity | 40 U insulin lispro once versus placebo (cross-over) during pancreatic clamp | No suppression of endogenous glucose production (in contrast to previous experiment with the same protocol in lean men) |
| Schriever et al. [75] | 47 volunteers (20 females) | 160 U human insulin | Stronger hypothalamic insulin response in men carrying the major allele in *DUSP8* SNP rs2334499 (assessed by fMRI) |
| Rodriguez-Raecke et al. [76] | Healthy 14 female, 16 males | 40 U human insulin versus Placebo (cross-over) | Females' but not males' olfactory sensitivity for n-butanol was lower after intranasal insulin administration vs. placebo |
| Heni et al. [77] | 15 young, healthy men | 160 U human insulin versus placebo (cross-over) during hyperglycaemic glucose clamp | Intranasal insulin increases second phase insulin secretion in humans with good hypothalamic insulin responsiveness (assessed by fMRI) and in the leaner half of the study group |
| Wingrove et al. [78] | 16 healthy males | 160 U human insulin versus placebo (saline) (cross-over) | Decreases in regional cerebral blood flow in areas dense in insulin receptors (bilateral amygdala) (assessed by fMRI) |
| Ferreira de Sá et al. [79] | 62 healthy students (31 females) with insulin,<br>61 healthy students (32 females) with placebo | 160 U human insulin versus placebo (parallel-design, double-blind) | Intranasal insulin facilitates fear extinction processes |

| | | | |
|---|---|---|---|
| Plomgaard et al. [9] | 9 healthy males | 160 U human insulin versus placebo (cross-over) | no influence on whole-body or hepatic glucose production at low circulating insulin concentrations (fasting) |
| Edwin Thanarajah et al. [80] | 21 normal weight and 21 overweight men | 40 U, 100 U, 160 U human insulin versus placebo (four-fold cross-over) | dose-dependent effects of intranasal insulin on midbrain functional connectivity (assessed by fMRI) are modulated by systemic insulin sensitivity |
| Edwin Thanarajah et al. [81] | 36 normal weight or overweight men | 40 U, 100 U, 160 U human insulin versus placebo (four-fold cross-over) | dose-dependent improvement of olfactory threshold |
| Rodriguez-Raecke et al. [20] | 30 healthy young men | 40 U human insulin versus placebo (cross-over) | no significant effect on hedonic ratings of food pictures, calorie content of purchased food products (virtual mock supermarket), or cookie consumption |
| Rosenbloom et al. [82] | 12 patients with Down syndrome ( ≥ 35 years) | 20 U insulin glulisin versus saline (cross-over) | Intranasal glulisine was safe and well tolerated |
| Wingrove et al. [83] | 10 normal weight and 13 overweight men | 160 U human insulin versus placebo (cross-over) | reduced CBF in hippocampus, insula, putamen, parahippocampal gyrus and fusiform gyrus only in the overweight group (assessed by fMRI) |
| Roque et al. [84] | 115 male and female patients undergoing cardiac surgery (43 with type 2 diabetes) | 40 U, 80 U human insulin or saline (parallel-design) | No clinical important hypoglycaemia as a result of intranasal insulin during cardiac surgery |

| | | | |
|---|---|---|---|
| Gwizdala et al. [85] | 116 healthy young persons (72 females) | 20 U, 40 U, 60 U, 80 U, 100 U, 120 U insulin aspart or saline (parallel-design) before 20 min of exercise or sitting | Exercise was not associated with an increase in risk when combined with lower doses of intranasal insulin. Higher likelihood of level 1 hypoglycaemia after 100 U of nasal insulin aspart |
| Kullmann et al. [86] | 10 young normal weight men | 160 U human insulin versus placebo (cross-over) | greater [11C]-raclopride binding potential in the striatum, i.e. reduction in synaptic dopamine levels (assessed by PET MRI) |
| Krug et al. [87] | 16 young normal weight men treated with 3-day transdermal estradiol and 16 young normal weight men treated with transdermal placebo | 160 U human insulin versus placebo (cross-over) | insulin's acute cognitive impact in young men is limited and not robustly potentiated by estradiol |
| Schneider et al. [21] | 35 normal weight and 17 women with obesity | 160 U human insulin versus placebo (cross-over) in postprandial state | Insulin decreased palatable food intake when satiated by reducing food reward with stronger effect in women with obesity |
| Wagner et al. [88] | 60 participants (30 females), wide BMI and age ranges | 160 U human insulin versus placebo (cross-over) | Obesity and sex influenced insulin effects on the brain's visual food cue reactivity (assessed by fMRI) |
| Wingrove et al. [89] | 10 normal weight and 14 overweight men | 160 U human insulin versus placebo (cross-over) | Weight group-dependent insulin responses in response to sweet stimuli mainly in prefrontal cortex and midbrain (assessed by fMRI) |

| | | | |
|---|---|---|---|
| Tiedemann et al. [90] | 50 overweight adults (30 females) >50 years, before diet intervention or control condition | Baseline measurement. 160 U human insulin versus placebo (cross-over) | Brain insulin responsiveness (assessed by fMRI) predicted the response to 3-month caloric restriction |
| Wagner et al. [91] | 110 participants (54 females), wide BMI and age ranges | 160 U human insulin versus placebo (cross-over) | region-specific relationship between brain insulin responsiveness (assessed by fMRI), age and peripheral insulin sensitivity |
| Schumann et al. [92] | 26 normal weight young men | 40 U human insulin or placebo (parallel-design) | Enhanced brain activation during olfactory-visual stimulation |
| Omiya et al. [93] | 24 patients undergoing thoracic aneurysm repair surgery | 40 U, 80 U human insulin or saline (parallel-design) | No effect on blood or CSF glucose concentrations during thoracic aneurysm repair surgery |
| Hummel et al. [5] | 11 and 15 young natural cycling women (hyperinsulinemic euglycaemic clamp or fMRI, respectively) | 160 U human insulin versus placebo (cross-over) | Relative hypothalamic insulin resistance in the luteal cycle phase (assessed by fMRI). Significant effect of nasal insulin on peripheral insulin sensitivity only during the follicular cycle phase. |

This table summarises effects of the acute administration of insulin as nasal spray, studies on the chronic administration are not included. Parts of the table (summarizing studies before November 2017) are modified from [94].

## Supplementary References


1. Heni M, Wagner R, Kullmann S, et al (2017) Hypothalamic and Striatal Insulin Action Suppresses Endogenous Glucose Production and May Stimulate Glucose Uptake During Hyperinsulinemia in Lean but Not in Overweight Men. Diabetes 66(7):1797–1806. https://doi.org/10.2337/db16-1380
2. Benedict C, Brede S, Schiöth HB, et al (2011) Intranasal insulin enhances postprandial thermogenesis and lowers postprandial serum insulin levels in healthy men. Diabetes 60(1):114–118. https://doi.org/10.2337/db10-0329
3. Heni M, Kullmann S, Ketterer C, et al (2012) Nasal insulin changes peripheral insulin sensitivity simultaneously with altered activity in homeostatic and reward-related human brain regions. Diabetologia 55(6):1773–1782. https://doi.org/10.1007/s00125-012-2528-y
4. Heni M, Wagner R, Kullmann S, et al (2014) Central insulin administration improves whole-body insulin sensitivity via hypothalamus and parasympathetic outputs in men. Diabetes 63(12):4083–4088. https://doi.org/10.2337/db14-0477
5. Hummel J, Benkendorff C, Fritsche L, et al (2023) Brain insulin action on peripheral insulin sensitivity in women depends on menstrual cycle phase. Nat Metab 5(9):1475–1482. https://doi.org/10.1038/s42255-023-00869-w
6. Ott V, Lehnert H, Staub J, Wönne K, Born J, Hallschmid M (2014) Central nervous insulin administration does not potentiate the acute glucoregulatory impact of concurrent mild hyperinsulinemia. Diabetes. https://doi.org/10.2337/db14-0931
7. Dash S, Xiao C, Morgantini C, Koulajian K, Lewis GF (2015) Intranasal insulin suppresses endogenous glucose production in humans compared with placebo in the presence of similar venous insulin concentrations. Diabetes 64(3):766–774. https://doi.org/10.2337/db14-0685
8. Gancheva S, Koliaki C, Bierwagen A, et al (2015) Effects of intranasal insulin on hepatic fat accumulation and energy metabolism in humans. Diabetes 64(6):1966–1975. https://doi.org/10.2337/db14-0892
9. Plomgaard P, Hansen JS, Ingerslev B, et al (2018) Nasal insulin administration does not affect hepatic glucose production at systemic fasting insulin levels. Diabetes Obes Metab. https://doi.org/10.1111/dom.13615
10. Xiao C, Dash S, Stahel P, Lewis GF (2018) Effects of Intranasal Insulin on Endogenous Glucose Production in Insulin Resistant Men. Diabetes Obes Metab. https://doi.org/10.1111/dom.13289
11. Stockhorst U, de Fries D, Steingrueber H-J, Scherbaum WA (2011) Unconditioned and conditioned effects of intranasally administered insulin vs placebo in healthy men: a randomised controlled trial. Diabetologia 54(6):1502–1506. https://doi.org/10.1007/s00125-011-2111-y
12. Heni M, Wagner R, Willmann C, et al (2020) Insulin Action in the Hypothalamus Increases Second-Phase Insulin Secretion in Humans. Neuroendocrinology 110(11–12):929–937. https://doi.org/10.1159/000504551
13. Iwen KA, Scherer T, Heni M, et al (2014) Intranasal insulin suppresses systemic but not subcutaneous lipolysis in healthy humans. J Clin Endocrinol Metab 99(2):E246-251. https://doi.org/10.1210/jc.2013-3169
14. Heni M, Wagner R, Kullmann S, Preissl H, Fritsche A (2015) Response to Comment on Heni et al. Central Insulin Administration Improves Whole-Body Insulin Sensitivity via Hypothalamus and Parasympathetic Outputs in Men. Diabetes 2014;63:4083-4088. Diabetes 64(6):e8-9. https://doi.org/10.2337/db15-0209
15. Benedict C, Kern W, Schultes B, Born J, Hallschmid M (2008) Differential sensitivity of men and women to anorexigenic and memory-improving effects of intranasal insulin. J Clin Endocrinol Metab 93(4):1339–1344. https://doi.org/10.1210/jc.2007-2606
16. Hallschmid M, Higgs S, Thienel M, Ott V, Lehnert H (2012) Postprandial

administration of intranasal insulin intensifies satiety and reduces intake of palatable snacks in women. Diabetes 61(4):782–789. https://doi.org/10.2337/db11-1390
17. Santiago JCP, Hallschmid M (2017) Central Nervous Insulin Administration before Nocturnal Sleep Decreases Breakfast Intake in Healthy Young and Elderly Subjects. Frontiers in Neuroscience 11
18. Krug R, Benedict C, Born J, Hallschmid M (2010) Comparable Sensitivity of Postmenopausal and Young Women to the Effects of Intranasal Insulin on Food Intake and Working Memory. The Journal of Clinical Endocrinology & Metabolism 95(12):E468–E472. https://doi.org/10.1210/jc.2010-0744
19. Jauch-Chara K, Friedrich A, Rezmer M, et al (2012) Intranasal Insulin Suppresses Food Intake via Enhancement of Brain Energy Levels in Humans. Diabetes 61(9):2261–2268. https://doi.org/10.2337/db12-0025
20. Rodriguez-Raecke R, Sommer M, Brünner YF, Müschenich FS, Sijben R (2020) Virtual grocery shopping and cookie consumption following intranasal insulin or placebo application. Exp Clin Psychopharmacol 28(4):495–500. https://doi.org/10.1037/pha0000330
21. Schneider E, Spetter MS, Martin E, et al (2022) The effect of intranasal insulin on appetite and mood in women with and without obesity: an experimental medicine study. Int J Obes (Lond) 46(7):1319–1327. https://doi.org/10.1038/s41366-022-01115-1
22. Hallschmid M, Benedict C, Schultes B, Born J, Kern W (2008) Obese men respond to cognitive but not to catabolic brain insulin signaling. Int J Obes (Lond) 32(2):275–282. https://doi.org/10.1038/sj.ijo.0803722
23. Bohringer A, Schwabe L, Richter S, Schachinger H (2008) Intranasal insulin attenuates the hypothalamic-pituitary-adrenal axis response to psychosocial stress. Psychoneuroendocrinology 33(10):1394–1400. https://doi.org/10.1016/j.psyneuen.2008.08.002
24. Feld GB, Wilhem I, Benedict C, et al (2015) Central Nervous Insulin Signaling in Sleep-Associated Memory Formation and Neuroendocrine Regulation. Neuropsychopharmacology. https://doi.org/10.1038/npp.2015.312
25. Thienel M, Wilhelm I, Benedict C, Born J, Hallschmid M (2017) Intranasal insulin decreases circulating cortisol concentrations during early sleep in elderly humans. Neurobiology of Aging 54:170–174. https://doi.org/10.1016/j.neurobiolaging.2017.03.006
26. Kern W, Born J, Schreiber H, Fehm HL (1999) Central nervous system effects of intranasally administered insulin during euglycemia in men. Diabetes 48(3):557–563
27. Ketterer C, Heni M, Stingl K, et al (2014) Polymorphism rs3123554 in CNR2 reveals gender-specific effects on body weight and affects loss of body weight and cerebral insulin action. Obesity (Silver Spring) 22(3):925–931. https://doi.org/10.1002/oby.20573
28. Dhindsa S, Chemitiganti R, Ghanim H, et al (2018) Intranasal Insulin Administration Does Not Affect LH Concentrations in Men with Diabetes. Int J Endocrinol 2018:6170154. https://doi.org/10.1155/2018/6170154
29. Kullmann S, Veit R, Peter A, et al (2018) Dose-Dependent Effects of Intranasal Insulin on Resting-State Brain Activity. J Clin Endocrinol Metab 103(1):253–262. https://doi.org/10.1210/jc.2017-01976
30. Kern W, Born J, Schreiber H, Fehm HL (1999) Central nervous system effects of intranasally administered insulin during euglycemia in men. Diabetes 48(3):557–563. https://doi.org/10.2337/diabetes.48.3.557
31. Born J, Lange T, Kern W, McGregor GP, Bickel U, Fehm HL (2002) Sniffing neuropeptides: a transnasal approach to the human brain. Nat Neurosci 5(6):514–516. https://doi.org/10.1038/nn849
32. Reger MA, Watson GS, Frey WH, et al (2006) Effects of intranasal insulin on cognition in memory-impaired older adults: modulation by APOE genotype. Neurobiol Aging 27(3):451–458. https://doi.org/10.1016/j.neurobiolaging.2005.03.016
33. Bohringer A, Schwabe L, Richter S, Schachinger H (2008) Intranasal insulin

attenuates the hypothalamic-pituitary-adrenal axis response to psychosocial stress. Psychoneuroendocrinology 33(10):1394–1400. https://doi.org/10.1016/j.psyneuen.2008.08.002
34. Reger MA, Watson GS, Green PS, et al (2008) Intranasal insulin administration dose-dependently modulates verbal memory and plasma amyloid-beta in memory-impaired older adults. J Alzheimers Dis 13(3):323–331
35. Guthoff M, Grichisch Y, Canova C, et al (2010) Insulin modulates food-related activity in the central nervous system. J Clin Endocrinol Metab 95(2):748–755. https://doi.org/10.1210/jc.2009-1677
36. Krug R, Benedict C, Born J, Hallschmid M (2010) Comparable sensitivity of postmenopausal and young women to the effects of intranasal insulin on food intake and working memory. J Clin Endocrinol Metab 95(12):E468-472. https://doi.org/10.1210/jc.2010-0744
37. Stingl KT, Kullmann S, Guthoff M, Heni M, Fritsche A, Preissl H (2010) Insulin modulation of magnetoencephalographic resting state dynamics in lean and obese subjects. Front Syst Neurosci 4:157. https://doi.org/10.3389/fnsys.2010.00157
38. Benedict C, Brede S, Schiöth HB, et al (2011) Intranasal Insulin Enhances Postprandial Thermogenesis and Lowers Postprandial Serum Insulin Levels in Healthy Men. Diabetes 60(1):114–118. https://doi.org/10.2337/db10-0329
39. Fan X, Copeland PM, Liu EY, et al (2011) No effect of single-dose intranasal insulin treatment on verbal memory and sustained attention in patients with schizophrenia. J Clin Psychopharmacol 31(2):231–234. https://doi.org/10.1097/JCP.0b013e31820ebd0e
40. Guthoff M, Stingl KT, Tschritter O, et al (2011) The insulin-mediated modulation of visually evoked magnetic fields is reduced in obese subjects. PLoS ONE 6(5):e19482. https://doi.org/10.1371/journal.pone.0019482
41. Stockhorst U, de Fries D, Steingrueber H-J, Scherbaum WA (2011) Unconditioned and conditioned effects of intranasally administered insulin vs placebo in healthy men: a randomised controlled trial. Diabetologia 54(6):1502–1506. https://doi.org/10.1007/s00125-011-2111-y
42. Stein MS, Scherer SC, Ladd KS, Harrison LC (2011) A randomized controlled trial of high-dose vitamin D2 followed by intranasal insulin in Alzheimer’s disease. J Alzheimers Dis 26(3):477–484. https://doi.org/10.3233/JAD-2011-110149
43. Grichisch Y, Çavuşoğlu M, Preissl H, et al (2012) Differential effects of intranasal insulin and caffeine on cerebral blood flow. Hum Brain Mapp 33(2):280–287. https://doi.org/10.1002/hbm.21216
44. Hallschmid M, Higgs S, Thienel M, Ott V, Lehnert H (2012) Postprandial administration of intranasal insulin intensifies satiety and reduces intake of palatable snacks in women. Diabetes 61(4):782–789. https://doi.org/10.2337/db11-1390
45. Jauch-Chara K, Friedrich A, Rezmer M, et al (2012) Intranasal insulin suppresses food intake via enhancement of brain energy levels in humans. Diabetes 61(9):2261–2268. https://doi.org/10.2337/db12-0025
46. Ketterer C, Heni M, Stingl K, et al (2014) Polymorphism rs3123554 in CNR2 reveals gender-specific effects on body weight and affects loss of body weight and cerebral insulin action. Obesity (Silver Spring) 22(3):925–931. https://doi.org/10.1002/oby.20573
47. Brünner YF, Benedict C, Freiherr J (2013) Intranasal insulin reduces olfactory sensitivity in normosmic humans. J Clin Endocrinol Metab 98(10):E1626-1630. https://doi.org/10.1210/jc.2013-2061
48. Kullmann S, Frank S, Heni M, et al (2013) Intranasal insulin modulates intrinsic reward and prefrontal circuitry of the human brain in lean women. Neuroendocrinology 97(2):176–182. https://doi.org/10.1159/000341406
49. Heni M, Wagner R, Kullmann S, et al (2014) Central insulin administration improves whole-body insulin sensitivity via hypothalamus and parasympathetic outputs in men.

Diabetes 63(12):4083–4088. https://doi.org/10.2337/db14-0477
50. Iwen KA, Scherer T, Heni M, et al (2014) Intranasal insulin suppresses systemic but not subcutaneous lipolysis in healthy humans. J Clin Endocrinol Metab 99(2):E246-251. https://doi.org/10.1210/jc.2013-3169
51. Novak V, Milberg W, Hao Y, et al (2014) Enhancement of vasoreactivity and cognition by intranasal insulin in type 2 diabetes. Diabetes Care 37(3):751–759. https://doi.org/10.2337/dc13-1672
52. Zhang H, Hao Y, Manor B, et al (2015) Intranasal insulin enhanced resting-state functional connectivity of hippocampal regions in type 2 diabetes. Diabetes 64(3):1025–1034. https://doi.org/10.2337/db14-1000
53. Ferreira de Sá DS, Schulz A, Streit FE, et al (2014) Cortisol, but not intranasal insulin, affects the central processing of visual food cues. Psychoneuroendocrinology 50:311–320. https://doi.org/10.1016/j.psyneuen.2014.09.006
54. Schilling TM, Ferreira de Sá DS, Westerhausen R, et al (2014) Intranasal insulin increases regional cerebral blood flow in the insular cortex in men independently of cortisol manipulation. Hum Brain Mapp 35(5):1944–1956. https://doi.org/10.1002/hbm.22304
55. Brünner YF, Kofoet A, Benedict C, Freiherr J (2015) Central insulin administration improves odor-cued reactivation of spatial memory in young men. J Clin Endocrinol Metab 100(1):212–219. https://doi.org/10.1210/jc.2014-3018
56. Gancheva S, Koliaki C, Bierwagen A, et al (2015) Effects of intranasal insulin on hepatic fat accumulation and energy metabolism in humans. Diabetes 64(6):1966–1975. https://doi.org/10.2337/db14-0892
57. Kullmann S, Heni M, Veit R, et al (2017) Intranasal insulin enhances brain functional connectivity mediating the relationship between adiposity and subjective feeling of hunger. Sci Rep 7(1):1627. https://doi.org/10.1038/s41598-017-01907-w
58. Kullmann S, Fritsche A, Wagner R, et al (2017) Hypothalamic insulin responsiveness is associated with pancreatic insulin secretion in humans. Physiol Behav 176:134–138. https://doi.org/10.1016/j.physbeh.2017.03.036
59. Kullmann S, Heni M, Veit R, et al (2015) Selective insulin resistance in homeostatic and cognitive control brain areas in overweight and obese adults. Diabetes Care 38(6):1044–1050. https://doi.org/10.2337/dc14-2319
60. Schöpf V, Kollndorfer K, Pollak M, Mueller CA, Freiherr J (2015) Intranasal insulin influences the olfactory performance of patients with smell loss, dependent on the body mass index: A pilot study. Rhinology 53(4):371–378. https://doi.org/10.4193/Rhin15.065
61. Feld GB, Wilhem I, Benedict C, et al (2016) Central Nervous Insulin Signaling in Sleep-Associated Memory Formation and Neuroendocrine Regulation. Neuropsychopharmacology 41(6):1540–1550. https://doi.org/10.1038/npp.2015.312
62. Brünner YF, Rodriguez-Raecke R, Mutic S, Benedict C, Freiherr J (2016) Neural correlates of olfactory and visual memory performance in 3D-simulated mazes after intranasal insulin application. Neurobiol Learn Mem 134 Pt B:256–263. https://doi.org/10.1016/j.nlm.2016.07.027
63. Hamidovic A, Khafaja M, Brandon V, et al (2017) Reduction of smoking urges with intranasal insulin: a randomized, crossover, placebo-controlled clinical trial. Mol Psychiatry. https://doi.org/10.1038/mp.2016.234
64. Hamidovic A, Candelaria L, Rodriguez I, Yamada M, Nawarskas J, Burge MR (2018) Learning and memory performance following acute intranasal insulin administration in abstinent smokers. Hum Psychopharmacol 33(2):e2649. https://doi.org/10.1002/hup.2649
65. Heni M, Wagner R, Kullmann S, et al (2017) Hypothalamic and Striatal Insulin Action Suppresses Endogenous Glucose Production and May Stimulate Glucose Uptake During Hyperinsulinemia in Lean but not in Overweight Men. Diabetes. https://doi.org/10.2337/db16-1380
66. Rodriguez-Raecke R, Yang H, Bruenner YF, Freiherr J (2017) Intranasal Insulin

Boosts Gustatory Sensitivity. J Neuroendocrinol 29(1). https://doi.org/10.1111/jne.12449
67. Santiago JCP, Hallschmid M (2017) Central Nervous Insulin Administration before Nocturnal Sleep Decreases Breakfast Intake in Healthy Young and Elderly Subjects. Front Neurosci 11:54. https://doi.org/10.3389/fnins.2017.00054
68. Akintola AA, van Opstal AM, Westendorp RG, Postmus I, van der Grond J, van Heemst D (2017) Effect of intranasally administered insulin on cerebral blood flow and perfusion; a randomized experiment in young and older adults. Aging (Albany NY) 9(3):790–802. https://doi.org/10.18632/aging.101192
69. Thienel M, Wilhelm I, Benedict C, Born J, Hallschmid M (2017) Intranasal insulin decreases circulating cortisol concentrations during early sleep in elderly humans. Neurobiol Aging 54:170–174. https://doi.org/10.1016/j.neurobiolaging.2017.03.006
70. Opstal AM van, Akintola AA, Elst M van der, et al (2017) Effects of intranasal insulin application on the hypothalamic BOLD response to glucose ingestion. Scientific Reports 7(1):13327. https://doi.org/10.1038/s41598-017-13818-x
71. Kullmann S, Veit R, Peter A, et al (2017) Dose dependent effects of intranasal insulin on resting-state brain activity. J Clin Endocrinol Metab. https://doi.org/10.1210/jc.2017-01976
72. Krug R, Mohwinkel L, Drotleff B, Born J, Hallschmid M (2018) Insulin and Estrogen Independently and Differentially Reduce Macronutrient Intake in Healthy Men. J Clin Endocrinol Metab 103(4):1393–1401. https://doi.org/10.1210/jc.2017-01835
73. Yokoyama H, Takeda R, Kawai E, et al (2018) Inhibitory Effects of Intranasal Administration of Insulin on Fat Oxidation during Exercise Are Diminished in Young Overweight Individuals. Journal of Clinical Medicine 7(10):308. https://doi.org/10.3390/jcm7100308
74. Dhindsa S, Chemitiganti R, Ghanim H, et al (2018) Intranasal Insulin Administration Does Not Affect LH Concentrations in Men with Diabetes. International Journal of Endocrinology 2018:1–7. https://doi.org/10.1155/2018/6170154
75. Schriever SC, Kabra DG, Pfuhlmann K, et al (2020) Type 2 diabetes risk gene Dusp8 regulates hypothalamic Jnk signaling and insulin sensitivity. J Clin Invest 130(11):6093–6108. https://doi.org/10.1172/JCI136363
76. Rodriguez-Raecke R, Brünner YF, Kofoet A, Mutic S, Benedict C, Freiherr J (2018) Odor Sensitivity After Intranasal Insulin Application Is Modulated by Gender. Frontiers in Endocrinology 9. https://doi.org/10.3389/fendo.2018.00580
77. Heni M, Wagner R, Willmann C, et al (2019) Insulin action in the hypothalamus increases second phase insulin secretion in humans. Neuroendocrinology. https://doi.org/10.1159/000504551
78. Wingrove J, Swedrowska M, Scherließ R, et al (2019) Characterisation of nasal devices for delivery of insulin to the brain and evaluation in humans using functional magnetic resonance imaging. Journal of Controlled Release 302:140–147. https://doi.org/10.1016/j.jconrel.2019.03.032
79. Ferreira de Sá DS, Römer S, Brückner AH, Issler T, Hauck A, Michael T (2020) Effects of intranasal insulin as an enhancer of fear extinction: a randomized, double-blind, placebo-controlled experimental study. Neuropsychopharmacology. https://doi.org/10.1038/s41386-019-0593-3
80. Edwin Thanarajah S, Iglesias S, Kuzmanovic B, et al (2019) Modulation of midbrain neurocircuitry by intranasal insulin. Neuroimage 194:120–127. https://doi.org/10.1016/j.neuroimage.2019.03.050
81. Thanarajah SE, Hoffstall V, Rigoux L, Hanssen R, Brüning JC, Tittgemeyer M (2019) The role of insulin sensitivity and intranasally applied insulin on olfactory perception. Sci Rep 9(1):1–8. https://doi.org/10.1038/s41598-019-43693-7
82. Rosenbloom M, Barclay T, Johnsen J, et al (2020) Double-Blind Placebo-Controlled Pilot Investigation of the Safety of a Single Dose of Rapid-Acting Intranasal Insulin in Down

Syndrome. Drugs R D. https://doi.org/10.1007/s40268-020-00296-2
83. Wingrove JO, O'Daly O, Forbes B, Swedrowska M, Amiel SA, Zelaya FO (2021) Intranasal insulin administration decreases cerebral blood flow in cortico-limbic regions: A neuropharmacological imaging study in normal and overweight males. Diabetes Obes Metab 23(1):175–185. https://doi.org/10.1111/dom.14213
84. Roque P, Nakadate Y, Sato H, et al (2021) Intranasal administration of 40 and 80 units of insulin does not cause hypoglycemia during cardiac surgery: a randomized controlled trial. Can J Anaesth 68(7):991–999. https://doi.org/10.1007/s12630-021-01969-5
85. Gwizdala KL, Ferguson DP, Kovan J, Novak V, Pontifex MB (2021) Placebo controlled phase II clinical trial: Safety and efficacy of combining intranasal insulin & acute exercise. Metab Brain Dis 36(6):1289–1303. https://doi.org/10.1007/s11011-021-00727-2
86. Kullmann S, Blum D, Jaghutriz BA, et al (2021) Central Insulin Modulates Dopamine Signaling in the Human Striatum. The Journal of Clinical Endocrinology & Metabolism 106(10):2949–2961. https://doi.org/10.1210/clinem/dgab410
87. Krug R, Beier L, Lämmerhofer M, Hallschmid M (2022) Distinct and Convergent Beneficial Effects of Estrogen and Insulin on Cognitive Function in Healthy Young Men. J Clin Endocrinol Metab 107(2):e582–e593. https://doi.org/10.1210/clinem/dgab689
88. Wagner L, Veit R, Fritsche L, et al (2022) Sex differences in central insulin action: Effect of intranasal insulin on neural food cue reactivity in adults with normal weight and overweight. Int J Obes (Lond) 46(9):1662–1670. https://doi.org/10.1038/s41366-022-01167-3
89. Wingrove J, O'Daly O, De Lara Rubio A, et al (2022) The influence of insulin on anticipation and consummatory reward to food intake: A functional imaging study on healthy normal weight and overweight subjects employing intranasal insulin delivery. Hum Brain Mapp 43(18):5432–5451. https://doi.org/10.1002/hbm.26019
90. Tiedemann LJ, Meyhöfer SM, Francke P, Beck J, Büchel C, Brassen S (2022) Insulin sensitivity in mesolimbic pathways predicts and improves with weight loss in older dieters. Elife 11:e76835. https://doi.org/10.7554/eLife.76835
91. Wagner L, Veit R, Kübler C, et al (2023) Brain insulin responsiveness is linked to age and peripheral insulin sensitivity. Diabetes Obes Metab. https://doi.org/10.1111/dom.15094
92. Schumann K, Rodriguez-Raecke R, Sijben R, Freiherr J (2023) Elevated insulin levels engage the salience network during multisensory perception. Neuroendocrinology. https://doi.org/10.1159/000533663
93. Omiya K, Nakadate Y, Schricker T (2023) Intranasal insulin and cerebrospinal fluid glucose levels. J Anesth 37(5):818–819. https://doi.org/10.1007/s00540-023-03233-0
94. Schmid V, Kullmann S, Gfrörer W, et al (2018) Safety of intranasal human insulin: a review. Diabetes Obes Metab. https://doi.org/10.1111/dom.13279
