# Supplementary material for: The insulin resistant brain: impact on whole-body metabolism and body fat distribution
Source: Diabetologia. 2024 Feb 16;67(7):1181–91. doi: 10.1007/s00125-024-06104-9 (PMC11153284; doi:10.1007/s00125-024-06104-9)
Supplement: Supplementary file 2 — Slideset of figures (PPTX 390 KB) [file 125_2024_6104_MOESM2_ESM.pptx]

## Slide 1
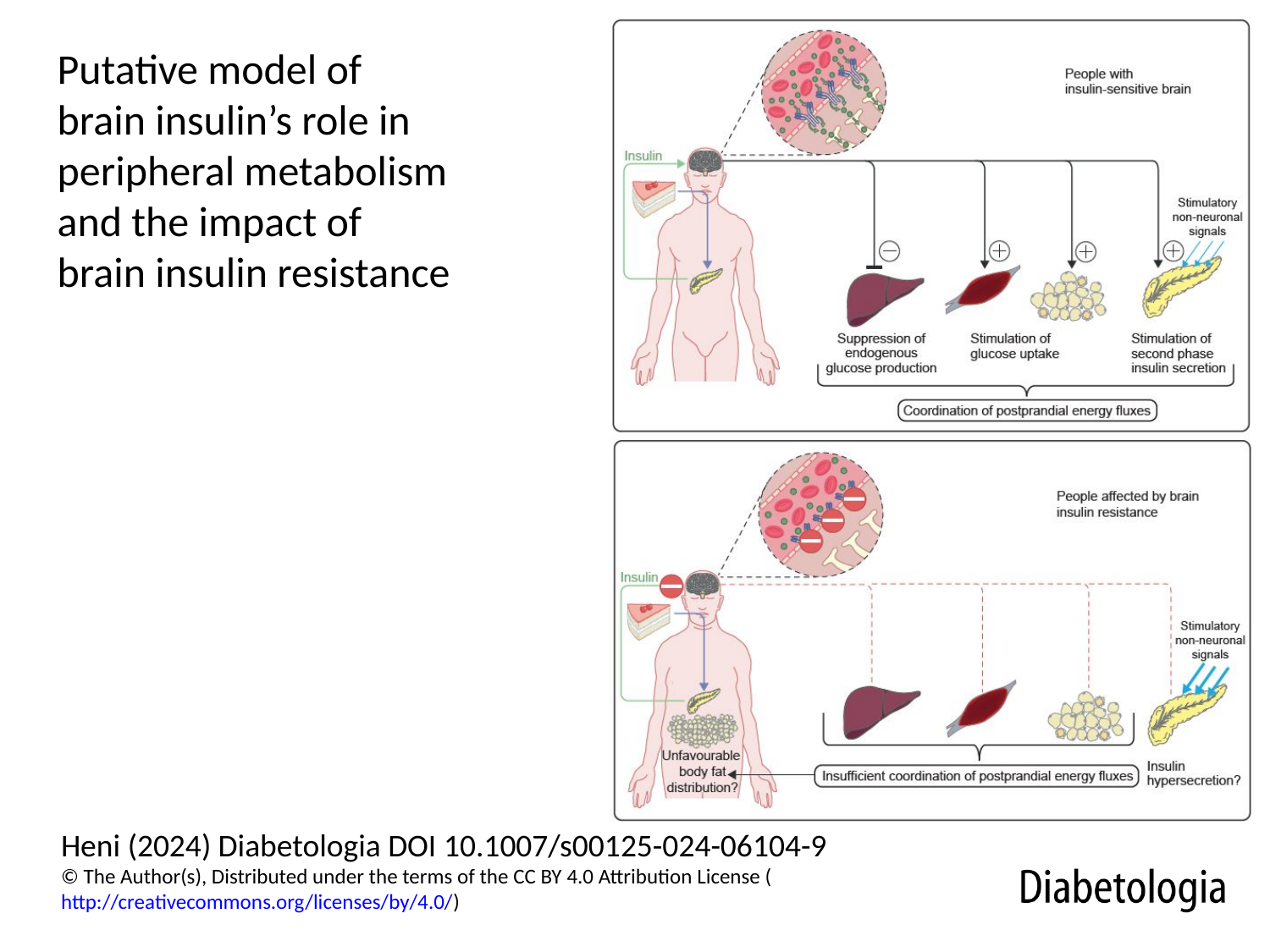

Putative model of brain insulin’s role in peripheral metabolism and the impact of brain insulin resistance
Heni (2024) Diabetologia DOI 10.1007/s00125-024-06104-9
© The Author(s), Distributed under the terms of the CC BY 4.0 Attribution License (http://creativecommons.org/licenses/by/4.0/)

## Slide 2
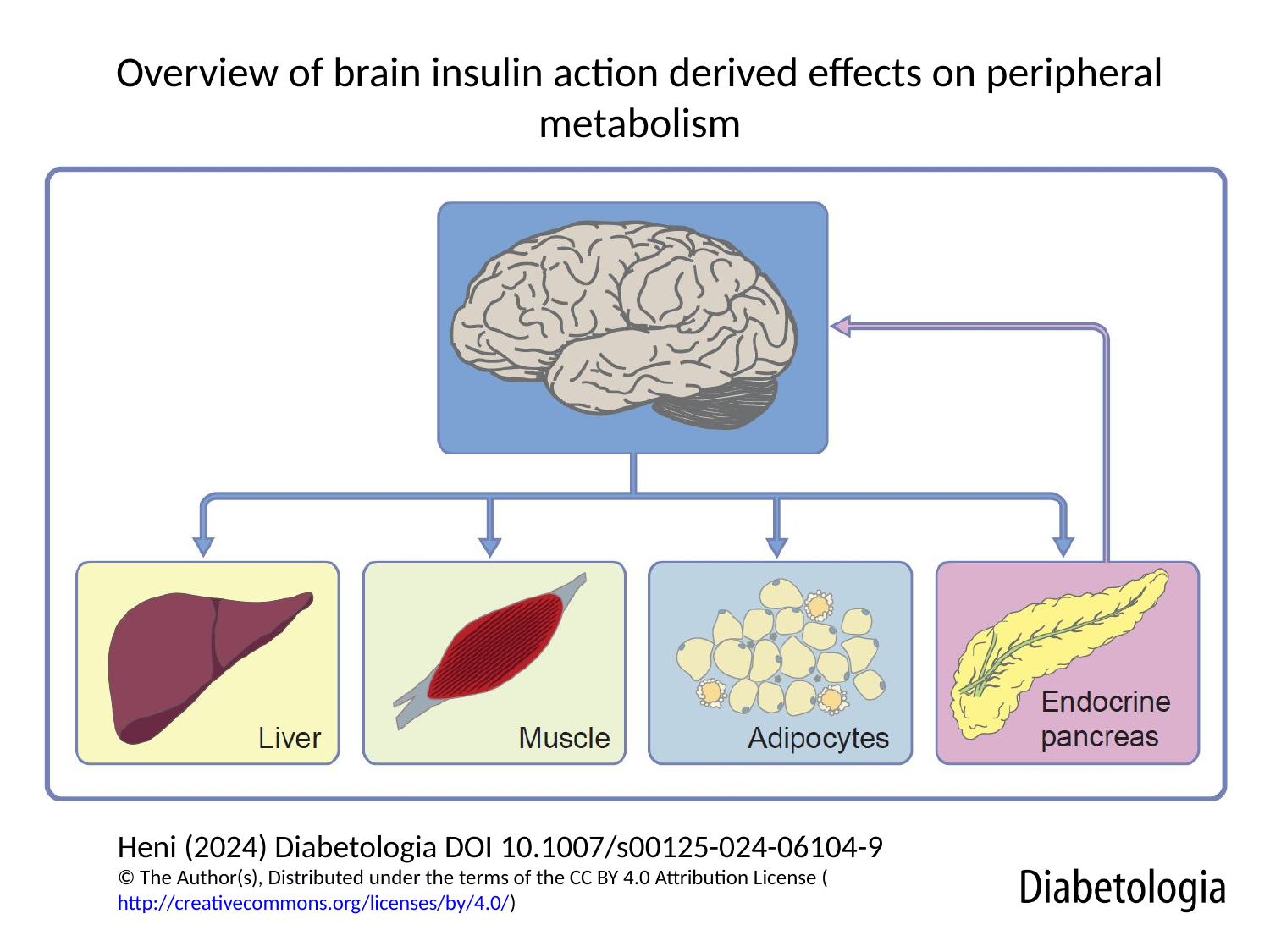

Overview of brain insulin action derived effects on peripheral metabolism
Heni (2024) Diabetologia DOI 10.1007/s00125-024-06104-9
© The Author(s), Distributed under the terms of the CC BY 4.0 Attribution License (http://creativecommons.org/licenses/by/4.0/)
